# Prediction of macrometastasis in axillary lymph nodes of patients with invasive breast cancer and the utility of the SUV lymph node/tumor ratio using FDG-PET/CT

**DOI:** 10.1186/s12957-014-0424-2

**Published:** 2015-02-14

**Authors:** Manabu Futamura, Takahiko Asano, Kazuhiro Kobayashi, Kasumi Morimitsu, Masahito Nawa, Masako Kanematsu, Akemi Morikawa, Ryutaro Mori, Kazuhiro Yoshida

**Affiliations:** Department of Breast and Molecular Oncology, Gifu University, Graduate School of Medicine, 1-1 Yanagido, Gifu, 501-1194 Japan; Department of Radiology, Gifu University, Graduate School of Medicine, 1-1 Yanagido, Gifu, 501-1194 Japan; Department of Tumor Pathology, Gifu University, Graduate School of Medicine, 1-1 Yanagido, Gifu, 501-1194 Japan; Department of Surgical Oncology, Gifu University, Graduate School of Medicine, 1-1 Yanagido, Gifu, 501-1194 Japan

**Keywords:** Axillary lymph node, Macrometastasis, Breast cancer, PET/CT, NT ratio

## Abstract

**Background:**

Axillary lymph node dissection (ALND) is important for improving the prognosis of patients with node-positive breast cancer. However, ALND can be avoided in select micrometastatic cases, preventing complications such as lymphedema or paresthesia of the upper limb. To appropriately omit ALND from treatment, evaluation of the axillary tumor burden is critical. The present study evaluated a method for preoperative quantification of axillary lymph node metastasis using positron emission tomography/computed tomography (PET/CT).

**Methods:**

The records of breast cancer patients who received radical surgery at the Gifu University Hospital (Gifu, Japan) between 2009 and 2014 were reviewed. The axillary lymph nodes were preoperatively evaluated by PET/CT. Lymph nodes were dissected by sentinel lymph node biopsy (SLNB) or ALND and were histologically diagnosed by experienced pathologists. The maximum standardized uptake value (SUVmax) was measured in both the axillary lymph node (SUV-LN) and primary tumor (SUV-T). The SUV-LN/T ratio (NT ratio) was calculated by dividing the SUV-LN by the SUV-T, and the efficacies of the NT ratio and SUV-LN were compared using receiver operating characteristic (ROC) curve analysis. The diagnostic performance was also compared between the techniques with the McNemar test.

**Results:**

A total of 171 operable invasive breast cancer patients were enrolled, comprising 69 node-positive patients (macrometastasis (Mac): n = 55; micrometastasis (Mic): n = 14) and 102 node-negative patients (Neg). The NT ratio for node-positive patients was significantly higher than in node-negative patients (0.5 vs. 0.316, respectively, *P* = 0.041). The NT ratio for Mac patients (0.571) was significantly higher than in Mic (0.227) and Neg (0.316) patients (*P* <0.01 and *P* = 0.021, respectively). The areas under the curves (AUCs) by ROC analysis for the NT ratio and SUV-LN were 0.647 and 0.811, respectively (*P* <0.01). In patients with an SUV-T ≥2.5, the modified AUCs for the NT ratio and SUV-LV were 0.757 and 0.797 (not significant).

**Conclusion:**

The NT ratio and SUV-LN are significantly higher in patients with axillary macrometastasis than in those with micrometastasis or no metastasis. The NT ratio and SUV-LN can help quantify axillary lymph node metastasis and may assist in macrometastasis identification, particularly in patients with an SUV-T ≥2.5.

**Electronic supplementary material:**

The online version of this article (doi:10.1186/s12957-014-0424-2) contains supplementary material, which is available to authorized users.

## Background

Axillary lymph node metastasis (ALNM) is an important prognostic factor for invasive breast cancer, and axillary lymph node dissection (ALND) may improve patient survival and decrease local recurrence [1,2]. Thus, ALND should be performed in patients with macrometastasis (Mac), as they have a poorer prognosis than patients with micrometastasis (Mic). Over half of patients with Mac develop non-sentinel lymph node (SLN) metastasis [3-6]; thus, the number of metastatic lymph nodes is critical in therapeutic planning, including chemo-endocrine therapy, surgery, and radiation therapy. ALND is important for disease control and breast cancer staging, but it may cause numerous complications such as lymph edema and sensory or motor disturbances in the upper limb. To precisely diagnose ALNM and perform minimally invasive ALND for breast cancer, sentinel lymph node biopsy (SLNB) is recommended in clinically node-negative (Neg) cases because it offers the same prognosis as ALND [7]. If the SLNB is positive, then ALND is required.

Even with additional irradiation or chemo-endocrine therapy, ALND does not improve the prognosis of patients with Mac [8,9]. In contrast, a minimally invasive procedure such as SLNB has fewer complications compared to ALND and is therefore commonly performed in clinically node-negative breast cancer patients. As indicated by the superior clinical outcomes in several clinical trials including the ACOSOG Z0011, IBCSG 23–01, and AATRM 048/13/2000 trials, small metastatic lesions that include Mic may not require additional ALND [9-11]. Thus, as therapy improves, ALND may be deemed unnecessary for select patients. To accomplish this goal, axillary evaluation is critical. ALND and SLNB provide postoperative clinical staging; therefore, preoperative diagnostic findings should be fully assessed in breast cancer patients. Ultrasonography (US), computed tomography (CT), and magnetic resonance imaging (MRI) are common diagnostic modalities, with sensitivities of 72% to 78%, 78%, and 67%, and specificities of 77% to 78%, 75%, and 78%, respectively [12-14]. Given their modest sensitivities and specificities, additional diagnostic tests are necessary as the diagnostic utility of these modalities is insufficient.

Recent therapeutic trends suggest that the tumor burden within axillary lymph nodes may impact surgical planning and prognosis; therefore, quantitative axillary assessment may be required to discriminate macrometastasis (Mac) from micrometastasis (Mic) and node-negative (Neg) cases. Common diagnostic modalities such as MRI, CT, and US can morphologically identify the metastatic lymph node, but quantification of the tumor burden and metastasis is difficult. Several methods of metastatic prediction have been described. Among them, nomogram is one representative predictive model used to identify sentinel or additional lymph node metastasis at several facilities. Nomogram is performed preoperatively and is based on clinicopathological data, including age, tumor size, location, lymphovascular invasion, and hormonal receptor activity in biopsy samples. However, the accuracy of nomogram is limited according to previous reports, with an area under the curve (AUC) in the range of 0.688 to 0.721 [15,16]. Recently, two methods of intraoperative detection were reported. The first, one-step nucleic acid amplification (OSNA), is a unique quantitative method that amplifies cytokeratin 19 messenger ribonucleic acid (mRNA). This method has quickly become widespread because it produces results similar to those of histopathologic staining with a >77.5% sensitivity and >95.8% specificity, and provides easy quantitative prediction [17,18]. The second technique, rapid double staining method with hematoxylin & eosin (HE) stain and immunohistochemistry, has decreased the false negative rate from 33.3% to 16.7% even in patients with Mic [19]. These methods can be useful in facilities possessing the required specialized equipment and pathology expertise, but also require greater concentration to perform due to time constraints.

In breast cancer medicine, fluorodeoxyglucose positron emission tomography/computed tomography (FDG-PET/CT) may be an acceptable alternative for detecting distant metastases [20,21]. In ALNM evaluation of breast cancer patients, PET/CT is less sensitive (20% to 37%) but more specific (>95%) than other modalities [22-25]. It also functionally detects abnormal glucose metabolism; a high maximum standard uptake value (SUVmax) indicates tumor activity within the axillary lymph node. For staging and estimating prognosis, the SUV Lymph node/tumor ratio (NT ratio), defined as the SUVmax ratio between the axillary lymph node (SUV-LN) and the primary tumor (SUV-T), is useful in detecting nodal malignancy in patients with non-small cell lung cancer [26].

Recent clinical trends indicate that additional lymphadenectomy is not required in patients with micrometastasis in the axillary lymph nodes; therefore, preoperative quantification of axillary disease is required to discriminate Mac from Mic and Neg. The present study investigates the utility of the NT ratio and SUV-LN as assessed by PET/CT for quantifying axillary lymph node metastasis in patients with invasive breast cancer.

## Methods

### Patients

ALNM was evaluated preoperatively using both PET/CT and conventional CT from June 2009 to February 2014 at Gifu University Hospital (Gifu, Japan). A total of 171 female breast cancer patients who underwent breast-conserving surgery (BCS) or mastectomy with either ALND or SLNB were enrolled in this retrospective study. Patients who were treated by neoadjuvant chemotherapy (NAC) and were pathologically diagnosed with positive lymph nodes by either biopsy or ALND were included. The dissected lymph nodes were histologically diagnosed by experienced pathologists. This study was approved by the Institutional Ethical Committee, and informed consent was obtained from all patients before their study inclusion.

### PET/CT and NT ratio

Whole body PET/CT (Biograph Sensation 16, Siemens Medical Solutions, Malvern, PA, USA) was performed within 1 month before treatment. All patients fasted at least 4 h prior to the PET/CT procedure. After the serum glucose concentration was confirmed as <150 mg/dL, patients were administered 185 MBq of 18F-FDG intravenously in the arm or leg contralateral to the primary breast tumor and rested quietly for 60 min before undergoing whole-body PET. CT was performed immediately after PET, and the PET/CT images were reconstructed. Two radiologists independently interpreted the PET/CT data. The FDG uptake in the primary tumor (SUV-T) and lymph node (SUV-LN) was semi-quantitatively analyzed using the SUVmax, which was calculated based on the measured activity, decay-corrected administrated dose, and patient weight. When calculating the SUV-LN, the axillary lymph node showing the highest SUV within the whole axillary space was selected. The NT ratio was calculated by dividing the SUV-LN by the SUV-T [26].

### Sentinel lymph node biopsy and axillary lymph node dissection

Each patient underwent either mastectomy or BCS based on the location and extension of the primary tumor. ALND was performed in patients with clinically positive axillary lymph nodes, while in clinically node-negative patients, SLNB was used for axillary evaluation [7]. At our institution, SLNB was performed using a dual-tracer technique, which is a combination of the blue dye method and the gamma probe-guided (RI) method. Imaging was performed 1 day preoperatively. Patients with PET/CT-positive axillae or pathologically SLNB-positive nodes underwent ALND.

### Pathological examination

The dissected sentinel lymph nodes were large enough for sectioning. The nodes were completely frozen intraoperatively or fixed in 10% formalin, and then embedded in paraffin and sectioned at 2-mm intervals. The lymph nodes that were excised by ALND were sectioned at the maximum diameter. Lesions were independently diagnosed as Mac (diameter >2 mm), Mic (0.2 mm < diameter ≤2.0 mm), isolated tumor cell (ITC; diameter ≤0.2 mm) [27,28], or no metastasis by two experienced pathologists based on microscopic examination of the HE stained sections. ITCs were categorized into the no metastasis group because ITC is considered clinically node-negative.

### Statistical analysis

All statistical analyses were performed using StatFlex version 6 (Osaka, Japan). Results presented as frequencies or percentages were analyzed as the mean ± standard deviation (SD). The NT ratio and SUV-LN results were compared by the Student’s *t* test. The sensitivity, specificity, positive predictive value (PPV), negative predictive value (NPV), and accuracy were estimated using the appropriate proportions, and the 95% confident intervals (CIs) were calculated using the Wilson score method [29]. Receiver operating characteristic (ROC) analysis was performed to determine the diagnostic utility of the NT ratio and SUV-LN in all enrolled patients and in those with an SUV-T ≥2.5. The diagnostic performance was evaluated using the McNemar test [30].

## Results

### Patient characteristics

The characteristics of the enrolled patients are detailed in Table 1. The mean age was 59.2 years, and the mean tumor size was 20.8 mm. Patients were staged as follows: Stage I disease: n = 60 (35.1%); Stage IIA: n = 60 (35.1%); Stage IIB: n = 34 (19.9%); Stage IIIA: n = 7 (4.1%); Stage IIIB: n = 5 (2.9%); and Stage IIIC: n = 3 (1.7%). Two patients were not staged because the invasive tumor tissue was lost during the diagnostic biopsy; these two lesions each measured approximately 20 mm maximally. The tumors were histologically graded as follows: grade I, n = 60 (35.1%); grade II, n = 40 (23.4%); and grade III, n = 69 (40.3%). Lymph node metastasis was verified using SLNB in 101 patients (59.1%) and ALND in 70 patients (40.9%). Histopathological evaluation was as follows: Mac, 55 patients (32.2%); Mic, 14 patients (8.2%); ITC, 5 patients (2.9%); and negative metastasis (Neg), 97 patients (56.7%). Lymph nodes with Mac and Mic (n = 69, 40.4%) were considered ALNM-positive, and ITC and negative cases were considered ALNM-negative (n = 102, 59.6%).

**Table 1:** **Patient characteristics (n = 171)**

	**Number**	**(%)**	**Mean ± SD**
Age (years)			59.2 ± 14.1
Tumor size (mm)			20.8 ± 11.6
*Stage*			
I	60	35.1	
IIA	60	35.1	
IIB	34	19.9	
IIIA	7	4.1	
IIIB	5	2.9	
IIIC	3	1.7	
ND	2	1.2	
*Histological grade*			
I	60	35.1	
II	40	23.4	
III	69	40.3	
ND	2	1.2	
*Verification of lymph node metastasis*			
Sentinel lymph node biopsy	101	59.1	
Axillary lymph node dissection	70	40.9	
*Histology of axillary lymph node*			
Macrometastasis	55	32.2	
Micrometastasis	14	8.2	
ITC	5	2.9	
Negative	97	56.7	

ITC: Isolated tumor cells; ND: Not determined; SD: Standard division.

### Analysis of lymph node metastasis by PET/CT

The utility of the NT ratio in estimating ALNM was determined based on the pathologic diagnosis. As shown in Table 2, the tumor size, SUV-T, and SUV-LN were significantly higher in the ALNM-positive cases than in the ALNM-negative cases (tumor size: 23.9 mm and 18.7 mm, *P* <0.01; SUV-T: 6.165 and 3.954, *P* <0.01; and SUV-LN: 2.289 and 0.841, *P* <0.01, respectively). The NT ratio differed significantly between ALNM-positive (0.5) and ALNM-negative (0.316) cases, respectively (*P* = 0.041; Table 2).

**Table 2:** **Axillary lymph node evaluation by PET/CT (mean ± SD)**

	**Metastasis-positive**	**Negative (+ITC)**
	**(n = 69)**	**(n = 102)**
Age (years)	56.7 ± 13.6	61.7 ± 14.4
Tumor size (mm)	23.9 ± 12.6^a^	18.7 ± 10.4
SUV-LN	2.289 ± 2.425^a^	0.841 ± 0.288
SUV-T	6.165 ± 3.871^a^	3.954 ± 3.524
NT ratio	0.5 ± 0.707^b^	0.316 ± 0.209

Patients with metastasis-positive nodes (n = 69) were compared to those with metastasis-negative nodes (n = 102). SD: Standard division. The node-negative group included those diagnosed with isolated tumor cells (ITC).

^a^ *P* <0.01.

^b^ *P* = 0.041 vs. the node-negative group.

**Figure 1:**
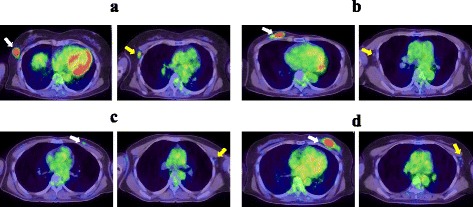
**Representative PET/CT images showing the NTcratio. (a)** True positive case, 57-year-old woman with an SUV-T of 8.69, SUV-LN of 3.11, and a NT ratio of 0.381. **(b)** True negative case, 63-year-old woman with an SUV-T of 9.63, SUV-LN of 1.07, and a NT ratio of 0.111. **(c)** False positive case, 42-year-old woman with an SUV-T of 1.27, SUV-LN of 1.69, and a NT ratio of 0.751. **(d)** False negative case, 62-year-old woman with an SUV-T of 10.88, SUV-LN of 1.45, and a NT ratio of 0.132. The primary tumor (right panel, yellow arrow) and axillary lymph node (left panel, white arrow) showing the highest SUV-LN in the axillae are indicated.

The ALNM cases were then classified into two groups: Mac (n = 55) and Mic (n = 14). The SUV-LN of the Mac group (2.63) was significantly higher than that of the Mic (0.935, *P* <0.01) and Neg groups (0.841, *P* <0.01). The NT ratio of the Mac group (0.571) was also higher than that of the Mic (0.227, *P* <0.01) and Neg (0.316, *P* = 0.021) groups. Furthermore, the NT ratio of the Mac group was higher than the NT ratio from both the Mic and Neg (0.306, *P* = 0.0155) groups. However, there was no statistical difference between the Mic and Neg groups in the NT ratio (Table 3). Representative cases are shown in Figure 1. These results suggest that the SUV-LN and NT ratio are helpful in predicting Mac in axillary lymph nodes.

**Table 3:** **Comparison of axillary lymph node evaluation results according to the metastases size (mean ± SD)**

	**Macrometastasis**	**Micrometastasis**	**Negative (+ITC)**	**Micrometastasis + Negative**
	**(n = 55)**	**(n = 14)**	**(n = 102)**	**(n = 116)**
Age (years)	56.6 ± 13.1	58.1 ± 17.6	61.7 ± 14.4	60.9 ± 14.5
Tumor size (mm)	24.5 ± 13.6^a^	20.9 ± 7.2	18.7 ± 10.4	19.0 ± 10.1
SUV-LN	2.63 ± 2.607^b^	0.935 ± 0.314	0.841 ± 0.288	0.856 ± 0.293
SUV-T	6.298 ± 4.122^c^	5.487 ± 2.223	3.954 ± 3.524	4.125 ± 3.427
NT ratio	0.571 ± 0.776^d^	0.227 ± 0.104	0.316 ± 0.209	0.306 ± 0.205

PET/CT parameters were compared between the four groups as follows: macrometastasis (Mac: n = 55), micrometastasis (Mic: n = 14), negative (Neg: n = 102), and micrometastasis and negative (Mic + Neg: n = 116). ^a^vs. the Neg group (*P* <0.01); ^b^vs. the Mic + Neg groups (*P* <0.01); ^c^vs. the Neg group (*P* <0.01) or the Mic + Neg group (*P* <0.01); ^d^vs. the Mic group (*P* <0.01), Neg group (*P* = 0.021), or the Mic + Neg groups (*P* = 0.0155).

### ROC curve analysis

**Figure 2:**
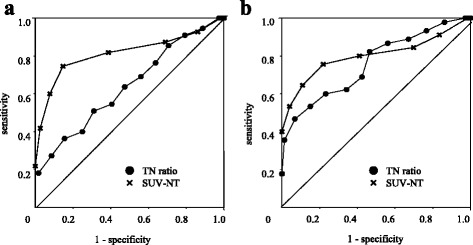
**ROC curves for the NT ratio and SUV-LN. (a)** Comparison of the AUCs between patients with Mac and those with Mic or negative nodes (Mic + Neg) in the patient population. The AUCs for the NT ratio and SUV-LN were 0.647 and 0.811, respectively (*P* <0.01). **(b)** AUCs comparing the Mac and Mic + Neg patients with an SUV-T ≥2.5 (n = 131). The AUCs for the NT ratio and SUV-LN were 0.757 and 0.797, respectively (*P* = 0.55, not significant).

A ROC curve analysis was performed to determine an ideal cutoff value and associated sensitivity and specificity for detecting Mac. An elevated SUVmax within the axillary lymph node appeared to indicate metastasis; therefore, the NT ratio and SUV-LN were compared by ROC. The analysis revealed that the AUC for the NT ratio (0.585) was inferior to that for the SUV-LN (0.779) in detecting metastasis (*P* <0.01). Under these conditions, the optimal cutoffs were 0.288 for the NT-ratio and 1.00 for the SUV-LN. Using the AUC, Mac cases were segregated from the remaining cases at an NT ratio of 0.647 and an SUV-LN of 0.811 (*P* <0.01; Figure 2a). However, in patients with an SUV-T <2.5, the NT ratio was 0.798, which was higher than the NT ratio (0.178) in patients with an SUV-T ≥2.5, despite the absence of metastasis.

Accordingly, the NT ratio and SUV-LN AUC were recalculated in patients with an SUV-T ≥2.5 (n = 118). There was no statistical difference in the AUC between the NT ratio and SUV-LN groups (0.757 and 0.797, respectively, *P* = 0.55). The optimal cutoff values were 0.199 for the NT ratio and 1.00 for the SUV-LN (Figure 2b). These results indicate that the NT ratio may be useful in detecting Mac, particularly in patients with an SUV-T ≥2.5.

### Comparison of diagnostic performance

Not only common modalities such as US, CT, or MRI, but also combined PET/CT have been suggested as useful in detecting axillary lymph node metastasis based on the SUV-LN. The characteristics of the NT ratio were determined by comparing its diagnostic performance to that of the SUV-LN. Using the set cutoff values (SUVmax: 1.00; NT ratio: 0.288), the NT ratio and SUV-LN sensitivities were 58.2% and 78.2%, their specificities were 59.5% and 80.2%, and their accuracies were 59.1% and 81.9%, respectively (Table 4). The NT ratio and SUV-LN differed significantly (*P* = 0.039). However, in the Neg group (n = 102), 71.2% of patients with a low SUV-T (SUVmax <2.5; n = 42) showed a high NT ratio (>0.4), which was only observed in 1.7% of patients with a high SUV-T (SUVmax ≥2.5; n = 60).

**Table 4:** **Comparison of diagnostic performance for detecting macrometastasis**

	**NT ratio**	**SUV-LN**	**Combination**
*All cases (n = 171)*	Cutoff 0.288	Cutoff : 1.00	
Sensitivity	58.2 (46.4-69.2)	78.2 (67.3-86.7)	85.5 (74.7-92.8)
Specificity	59.5 (50.0-68.5)	80.2 (75.0-84.2)	50.0 (44.9-53.5)
PPV	40.5 (29.6-52.2)	65.2 (56.1-72.2)	44.8 ( 39.1-48.6)
NPV	75.0 (64.9-83.4)	88.3 (83.0-92.5)	87.9 (78.9-94.0)
Accuracy	59.1 (51.5-66.2)	81.9 (75–87.3)	61.4 (54.5-66.1)
*SUV-T ≥ 2.5 (n = 118)*	Cutoff 0.199	Cutoff : 1.00	
Sensitivity	68.9 (56.4-79.6)	75.6 (63.5-85.1)	84.4 (72.7-92.6)
Specificity	65.8 (68.1-72.4)	76.7 (69.3-82.6)	54.8 (47.5-59.8)
PPV	55.4 (45.3-64.0)	65.4 (55.0-73.7)	53.5 (46.1-58.7)
NPV	77.4 (68.4-85.2)	83.6 (75.5-90.0)	85.1 (73.8-92.9)
Accuracy	66.9 (57.4-75.1)	76.3 (67.1-83.5)	66.1 (57.7-72.3)

The diagnostic performance of the NT ratio, SUV-LN, and both techniques in combination is indicated. Cutoff values for each modality were determined based on the sensitivity and specificity. In the whole patient population (n = 171), the cutoffs for the NT ratio and SUV-LN were 0.288 and 1.00, respectively; these values were compared using the McNemar test (*P* = 0.013). In patients with an SUV-T ≥2.5 (n = 118), the cutoffs for the NT ratio and SUV-LN were 0.199 and 1.00, respectively, with no significant difference noted.

Assuming that an NT ratio with a low SUV-T is unlikely to be clinically significant, patients with a high SUV-T (≥2.5; n = 118) were the focus of the remaining analysis. Using the set cutoff values (SUV max: 1.00; NT ratio: 0.199), the NT ratio and SUV-LN sensitivities were 68.9% and 75.6%, their specificities were 65.8% and 76.7%, and their accuracies were 66.9% and 76.3%, respectively. There was no statistical difference between the NT ratio and SUV-LN (*P* = 0.065). In addition, the positive predictive values (PPV) were 55.4% and 65.4%, and the negative predictive values (NPV) were 77.4% and 83.6%, respectively, and did not differ significantly. When the NT ratio and SUV-LN were used in combination, the sensitivity increased to 85.5% (for all cases) and 84.4% (cases with SUV-T ≥2.5), respectively.

## Discussion

For minimally invasive surgery of breast cancer, we strongly believe in the importance of preoperatively evaluating the axillary lymph node. We previously investigated the utility of navigation surgery based on composite PET/CT and US images, specifically in cases of axillary neoplasia, and found that PET/CT is a valuable tool for breast surgery [31]. The PET/CT-based NT ratio was selected for preoperative quantitative evaluation because SUV measurement is simple and easy to perform. Furthermore, this method can be used to evaluate both the SLN and the non-SLN in the entire axillary space. The technique was initially applied to non-small-cell lung cancer (NSCLC) as a universal predictor of mediastinal node malignancy and showed a 0.56 cutoff value, 94% sensitivity, and 72% specificity [26]. These previous data motivated us to use the NT ratio for axillary evaluation in breast cancer patients. Recently, one report described the utility of the NT ratio for predicting ALNM in breast cancer patients [32]. The mean NT ratio was 0.3, and the cutoff value was 0.2; when the diagnostic performance was assessed, the technique showed a 71.4% sensitivity and a 77.3% specificity. The AUC for the NT ratio was 0.776 and was superior to the AUC for the SUV-LN (0.705).

In the present study, the mean NT ratio for Mac (0.571) was significantly higher than observed for Mic (0.227) and node-negative lesions (0.316). Potentially, the NT ratio may reflect the tumor burden, though the number of Mic cases was small. Notably, 96% of patients with a high SUV-LN (≥1.5) had Mac, but a low SUV-LN (0.5-1.5) may interfere with evaluation. In 30 patients with Mac and a low SUV-LN, 17 (57%) had a high NT ratio (>0.288), suggesting that the NT-ratio may predict patients with Mac. However, node-negative cases may show false positive results, as shown in Figure 1. A total 42 of 46 (91%) node-negative cases with a low SUV-T (<2.5) showed a seemingly high NT ratio (mean: 0.54, range: 0.319-1.061). This discrepancy prompted a reexamination of the NT ratio accuracy in patients with a high SUV-T (≥2.5; n = 118), and no statistical difference was found between the AUCs of the NT-ratio (0.757) and SUV-LN (0.797; Figure 2b). Under these conditions, the sensitivities for Mac were 68.9% using the NT ratio and 75.6% using the SUV-LN. However, combining these methods increased the sensitivity to 84.4%. The NT ratio appears to be particularly reliable in patients with an SUV-T greater than 2.5. Our results suggest that the NT ratio is one option for the preoperative quantification of axillary lymph node metastasis. Combining the NT ratio and the SUV-LN may be important for minimizing false positive cases. However, tumors with a low SUV, including low-grade malignancies or benign inflammatory lesions, also warrant attention. Both morphological evaluation by conventional modalities and assessment by functional modalities such as the NT ratio and SUV-LN using PET/CT are required to quantitatively diagnose the axillary lymph node. Notably, all the data analyzed in this study were obtained in patients prior to treatment.

Whether the NT ratio is efficacious in patients receiving chemo, endocrine, and molecular target therapies is unclear. Reportedly, in tumors that were vulnerable to chemotherapy, the SUV-T significantly decreased after the second cycle of neoadjuvant chemotherapy, resulted in low sensitivity (66.7% to 68%) and high specificity (75% to 96.4%) for predicting the pathological complete response (pCR) [33,34]. At present, there are no data on lymph node assessment during neoadjuvant chemotherapy. The therapeutic response at the metastatic site, including lymph nodes, may differ from that for the primary tumor, which may make the NT ratio complex and difficult to understand. When evaluating the effect of neoadjuvant chemotherapy, changes in both the SUV-T and SUV-LN should be considered.

Axillary evaluation is crucial for staging and therapeutic planning in patients with invasive breast cancer. Although the current data was retrospectively analyzed at a single institution, our findings suggest that both the SUV-LN and the NT ratio obtained by PET/CT may help predict preoperative Mac in axillary lymph nodes. A prospective large cohort study is recommended to validate the NT ratio as a reliable predictor of Mac.

## Conclusion

To determine the need for axillary lymph node dissection in patients with invasive breast cancer, we diagnosed the presence of axillary lymph node metastasis using the NT ratio and SUV-LN obtained by PET/CT. Both the NT ratio and SUV-LN were significantly higher in patients with axillary macrometastasis than in those with micrometastasis or no metastasis. Although the utility of PET/CT in breast cancer remains unclear, the NT ratio appears to be helpful in quantifying axillary lymph node metastasis with similar utility to SUV-LN and can assist in macrometastasis identification, particularly in patients with an SUV-T greater than 2.5.
